# Effects of Chenpi (*Citrus reticulata* cv. *Chachiensis*) on serum antioxidant enzymes, inflammatory factors, and intestinal health in Beagle dogs

**DOI:** 10.3389/fmicb.2024.1415860

**Published:** 2025-01-07

**Authors:** Wencan Wang, Ling Xu, Yan Zhang, Yong Cao, Yixue Yang, Guo Liu, Xin Mao

**Affiliations:** ^1^Chongqing Sweet Pet Products Co., Ltd., Chongqing, China; ^2^Department of Animal Nutrition and Feed, College of Biological Engineering, Sichuan Water Conservancy Vocational College, Chengdu, China; ^3^Guangdong Provincial Key Laboratory of Nutraceuticals and Functional Foods, College of Food Science, South China Agricultural University, Guangzhou, China; ^4^College of Light Industry and Food Science, Zhongkai University of Agriculture and Engineering, Guangzhou, China

**Keywords:** Chenpi, intestinal flora, pet health, functional foods, dog

## Abstract

Ensuring companion animal welfare is a top priority for the pet industry and owners alike. The health of the pets can be directly and effectively improved through diet. Chenpi includes beneficial ingredients with proven anti-inflammatory, antioxidant, and immunomodulatory properties. The present investigation involved feeding snacks infused with Chenpi powder (CPP) to dogs for 42 days to examine the potential health benefits of CPP. The research evidenced a notable increase in serum superoxide dismutase (SOD), catalase (CAT), and glutathione peroxidase (GSH-Px) activity in dogs, accompanied by a decrease in malondialdehyde (MDA), interleukin-8 (IL-8), and interferon-gamma (IFN-γ) level. Additionally, CPP increased fecal scores and significantly reduced fecal odors due to inhibition of 3-methylindole, hydrogen sulfide (H_2_S), and ammonia nitrogen (NH_4_^+^-N), and also raised the levels of fecal secretory immunoglobulin A (SIgA). Analysis of the microbial composition *via* 16S rRNA sequencing showed that CPP increased *Bacteroidota* and decreased *Firmicutes* in the gut flora at the phylum level. Functional prediction study of microbial communities also showed that the CPP group enriched metabolic and genetic information processing pathways. In addition, there were significant correlations between serum indicators and several significantly altered microorganisms. These findings suggest that CPP can potentially enhance the overall health of dogs by reducing fecal odorants, enhancing antioxidant and immunological capabilities, and modulating intestinal flora. This study establishes a solid scientific foundation regarding the application of CPP in functional pet foods.

## 1 Introduction

The intestinal tract serves as the primary site for nutrient absorption and functions as the body’s largest immune organ, playing a crucial role in defending against invading pathogens and other potentially harmful substances (Turner, 2009). Therefore, the intestinal tract is a crucial component of a pet’s overall wellbeing, and its condition significantly influences the organism’s health. Continuous contact with the external environment exposes the intestinal tract to various stressors, making intestinal ailments one of the most frequent clinical conditions in dogs. As the importance of pets in people’s lives grows, pet health, particularly intestinal health, has been an increasing concern for pet owners (Alessandri et al., 2020). Intestinal microorganisms play an indispensable role in maintaining the normal function of the intestinal barrier, stimulating the immune system, and combating pathogenic microbes (Thursby and Juge, 2017). In addition, it has been suggested that disturbances in gut microbial homeostasis can trigger neurological disorders through the brain-gut axis (Cryan and Dinan, 2012). Intestinal health issues in dogs can arise from several sources, including genetics, environment, nutrition, diet, and viral and bacterial infections. However, the primary cause of intestinal diseases is disturbances in the intestinal flora (Blake and Suchodolski, 2016). In dogs and cats, common intestinal diseases, such as inflammatory bowel disease, acute diarrhea, and acute hemorrhagic diarrhea, are associated with an elevated presence of harmful intestinal bacteria (Allenspach et al., 2010; Guard et al., 2019; Janeczko et al., 2008; Suchodolski et al., 2010; Suchodolski et al., 2012b). Therefore, improving intestinal flora may have a preventive effect on developing intestinal diseases in pets.

Chenpi, the peel of citrus fruits, is renowned for its production in Xinhui, China. As a substance with dual medicinal and culinary properties, Chenpi contains many valuable beneficial compounds like flavonoids (Huang R. et al., 2020) and essential oils (Tranchida et al., 2012). These compounds have antioxidant (Chen et al., 2017), anti-inflammatory (Zhao et al., 2019), and immunomodulatory (Kim et al., 2019) effects. It has been shown that citrus peel dietary fiber enhanced serum and liver antioxidant enzyme activities as well as reduced malondialdehyde (MDA) levels in oxidative stress model mice (Fu et al., 2024). Similarly, citrus peel extract (CPE) had an enhancing effect on the activity of serum antioxidant enzymes, such as superoxide dismutase (SOD) and catalase (CAT), while decreasing MDA levels in rabbits under heat stress (El-Gindy et al., 2023). Zhao et al. (2022) found that CPE also increased milk total antioxidant capacity (T-AOC) and SOD activity. On the other side, Kono et al. (2022) reported that nobiletin, a polymethoxylated flavone in Chenpi, inhibited NLR family pyrin domain containing 3 (*Nlrp3)*, interleukin-1β (*IL-1*β), and tumor necrosis factor-α (*TNF*-α) gene expression in bladder mucosa of cystitis mice. Moreover, the hesperetin in citrus peel significantly reduced NF-κB, TNF-α, and IL-1β protein levels in the cerebral cortex and hippocampus of LPS-induced mice (Muhammad et al., 2019). Leray et al. (2011) indicated that supplementation with CPE notably reduced *INF*-γ gene expression in peripheral blood mononuclear cells of obese cats and improved obesity-induced inflammatory status. Moreover, studies have demonstrated that citrus peel can alleviate colonic mucosal injury (Han et al., 2023; Kawabata et al., 2018), inhibit central nervous system inflammation and oxidative stress (Omasa et al., 2023), and prevent liver injury in mice (Chowdhury et al., 2015). Research has shown that nobiletin also has strong anti-tumor effects on lung, stomach, and colon cancer (Manthey and Guthrie, 2002; Rodriguez et al., 2002). The flavonoid extracts of Chenpi also have weight loss and lipid-lowering functions, which can reduce lipid accumulation in HepG2 cells and lower the levels of total cholesterol and triglycerides in the serum of mice (Kim et al., 2003; Su et al., 2019). It is noteworthy that citrus peel has the potential to enhance intestinal health and function. The citrus peel contains plentiful insoluble dietary fiber, which aids in regulating the composition, structure, and activity of intestinal flora, thus promoting the intestinal health of rats and mice (Huang J. Y. et al., 2020; Paturi et al., 2017). *In vivo* studies have also indicated that citrus peel can modify the composition and abundance of mice’s intestinal flora (Hu et al., 2021; Qian et al., 2021). Furthermore, citrus peel has been shown to modulate intestinal immunity and enhance the immunological balance of the intestinal mucosa in mice (Chau et al., 2005).

Currently, citrus peels are already being used in livestock feed. However, there is a shortage of research on its potential benefits for pet health. Pets, being domesticated animals, have frequent interaction with humans, and the wellbeing of the pet is interconnected with the physical and emotional wellbeing of the pet owners. Therefore, the present study aims to evaluate the impact of Chenpi powder (CPP) on antioxidant status, immunomodulation, and intestinal health of dogs. The research involves assessing serum antioxidant and immune indicators, fecal odorant content, and examining changes in fecal microorganisms through 16S rRNA sequencing after feeding dogs with CPP-containing snacks. The present study will provide a scientific basis for incorporating CPP into functional foods to enhance pet health.

## 2 Materials and methods

### 2.1 Study design

The CPP used in the current study was purchased from Lianfu Food Co., Ltd. (Taizhou, China). Based on previous experimental explorations, the experimental snacks with a CPP concentration of 0.2% were formulated based on the control snacks. The composition of the control snacks and CPP is shown in Tables 1, 2, respectively.

**TABLE 1:** Composition of the control snacks.

Ingredients	Content (g)
Corn starch	60.00
Calcium carbonate	0.80
Glycerol	10.00
Monoacylglyceride	0.80
Sodium hexametaphosphate	0.50
Sugar	1.00
Chicken meal	2.00
Chicken paste	4.00
Chicken liver meal	3.00
Potassium sorbate	0.20
Calcium propionate	0.08
Water	20.00
Total	102.38

**TABLE 2:** Composition of the CPP.

Ingredients	Content (μg/g)
Heptamethoxyflavone	33.58
Hesperidin	5,545.56
Quercetin	1.69
Narirutin	60.03
Hesperetin	2.45
Didymin	29.33
Nobiletin	22.65
Sinensetin	144.75

This study included 20 adult and healthy Beagles (10 male and 10 female). The control group (CON) consisted of five male and five female dogs with an average age of 2.35 ± 0.05 years, average body weight of 13.29 ± 0.30 kg and average body condition score (BCS) of 4.6 ± 0.16. The CPP group consisted of five male and five female dogs with an average age of 2.42 ± 0.06 years, average body weight of 12.92 ± 0.31 kg and average BCS of 4.7 ± 0.21. The kennel was kept at 25°C with a humidity of 55%, and all dogs were housed individually in cages (1.2 m length × 1.2 m width × 1.4 m height), ensuring sufficient light and exercise once a day. All dogs received two meals of maintenance diet (Jiangsu Xietong Inc., Nanjing, China; 5.9% ash, 20.88% protein, 8.4% fat, and 3.5% fiber) per day, 150 g per meal at 9:00 a.m. and 3:00 p.m., and with adequate water intake. Two hours after consuming the maintenance diet, the dogs in the CON and the CPP group were provided 30 g of snacks, respectively. Prior to the experiment, all dogs were acclimated to the snacks for 7 days. Afterward, serum biochemical analysis was conducted to evaluate the health status of the dogs. This study lasted for 42 days, and all the procedures were approved by the Institutional Animal Care and Use Committee of South China Agriculture University (Permit Number: SCAU-AEC-2010-0416).

### 2.2 Serum biochemical analysis

Canine blood samples were collected using previously established procedures with some modifications (Wang et al., 2022). Briefly, the blood samples were collected on the morning of day 0 (1 day before the experiment) and day 42 in a fasting state. Firstly, the dogs were secured in a suitable position, and the hair at the blood collection site was removed. Next, the forelimbs were tightly wrapped with tape to constrict the veins, and a syringe was used to extract blood gradually following the sterilization of the skin using 75% alcohol. A volume of 3 ml of blood was obtained and then transferred into sterile EP tubes. The tubes were centrifuged at 1,200 × g for 15 min at 4°C to separate the serum. A portion of the serum was promptly applied for the automatic biochemical assessment (Seamaty, SMT-120VP, Chengdu, China). In contrast, the remaining serum was stored at −20°C for subsequent determination of serum SOD (Solarbio Science & Technology Co., Ltd., Beijing, China), CAT (Solarbio Science & Technology Co., Ltd., Beijing, China), glutathione peroxidase (GSH-Px) (UpingBio, Shenzhen, China), MDA (Jiancheng Bioengineering Institute, Nanjing, China), and enzyme-linked immunosorbent assay (ELISA) testing.

### 2.3 Measurement of fecal odor substances

Dogs in the morning fasting state on days 0 and 42, fecal samples were collected and subjected to fecal scoring. After natural defecation, dogs’ feces were scored according to the method by Middelbos et al. (2007). The criteria were as follows: 1 = hard, dry pellets; small, hard mass; 2 = hard-formed, dry stool; remains firm and soft; 3 = soft, formed, and moist stool, retains shape; 4 = soft, unformed stool; assumes the shape of unformed stool; assumes the shape of the container; and 5 = watery; liquid that can be poured. After scoring, feces not contaminated with urine and hair were collected with a sterile swab, transferred to 15 ml sterile EP tubes, and immediately kept on dry ice (−78.5°C). The fecal content of 3-methylindole was measured by gas chromatography/mass spectrometry (GC/MS; GC2010-QP2010, Shimadzu, Tokyo, Japan). Additionally, the fecal levels of hydrogen sulfide (H_2_S) and ammonia nitrogen (NH_4_^+^-N) were determined using specific kits (UpingBio, Shenzhen, China).

### 2.4 ELISA analysis

The content of canine fecal secretory immunoglobulin A (SIgA) (F66008-A), serum IL-1β (F5558-A), TNF-α (F7578-A), IL-8 (F4427-A), and interferon-gamma (IFN-γ) (F66003-A) was measured using the respective ELISA kits (Fankew, Shanghai Kexing Trading Co., Ltd., Shanghai, China).

### 2.5 Fecal microbiota sequencing analysis

#### 2.5.1 Fecal DNA extraction, amplification, and sequencing

Fecal microbial DNA was extracted using the Stool DNA Kit (Tiangen Biotech, Beijing, China). The variable regions of V3–V4 in 16S rRNA genes were amplified *via* primers pair 341F (5′-CCTAYGGGRBGCASCAG-3′) and 806R (5′-GGACTACNNGGGTATCTAAT-3′). Briefly, PCR amplification was performed by combining 15 μl of Phusion^®^ High-Fidelity PCR Master Mix (New England Biolabs, Ipswich, MA, USA), 0.2 μM of primers, and 10 ng of DNA template. The PCR conditions were as follows: pre-denaturation at 98°C for 1 min followed by 30 cycles at 98°C (10 s), 50°C (30 s), and 72°C (30 s). Finally, 72°C was kept for 5 min to obtain the PCR products, and it was purified using the magnetic beads. Based on the concentration of products, an equal amount of sample was fully mixed and the products were electrophoresed to recover the target bands. Sequencing libraries were constructed using the NEBNext Ultra II DNA Library Prep Kit (New England Biolabs, Ipswich, MA, USA). The libraries were examined using Qubit (Thermo Fisher Scientific, Waltham, MA, USA) and q-PCR, then complied with the high-throughput sequencing using the No-vaSeq6000 PE250 (Novogene Co., Ltd., Beijing, China).

#### 2.5.2 Bioinformatics analysis

The barcode and primer sequences were truncated to get the paired-end reads. These reads were then joined together using the FLASH program (v 1.2.11^1^) for each sample, producing raw tags (Magoc and Salzberg, 2011). Next, the reverse primer sequence was matched using Cutadapt, and the remaining sequence was cut off to prevent it from interfering with subsequent analysis (Martin, 2011). The Fastp program (v 0.23.1) was utilized to eliminate the spliced raw tags and generate purified tags (Bokulich et al., 2013). The final effective tags were generated by comparing the tags obtained from the foregoing approach with the species annotation database^2^ for detecting and removing chimeric sequences (Edgar et al., 2011). The denoising process in the QIIME2 software (v 2022.2) utilizing the DADA2 plugin (Wang et al., 2021) resulted in the acquisition of the final amplicon sequence variants (ASVs) and feature table. Following that, species annotation was conducted utilizing the Silva database.

Venn diagrams were generated using the Venn Diagram function in *R* software (v 4.2.1). Based on the top 10 species concerning the abundance at various taxonomic levels, the SVG function was used to plot relative abundance distribution in Perl as a histogram for each sample. The QIIME2 was utilized to calculate alpha diversity indices, such as Observed_Operational Taxonomic Units (OTUs), Shannon, Chao1, and Simpson. Beta diversity was assessed using the principle coordinate analysis (PCoA) in QIIME2. Moreover, the bacterial composition disparity between the two groups was further investigated utilizing ANOSIM to discern significant differences. The linear discriminant analysis (LDA) effect size (LEfSe) was used to identify the major microbial taxa that differed significantly in the experimental group. Furthermore, the *t*-test (*p*-value < 0.05) was applied for statistical analysis, and the microbial functions were predicted using PICRUSt analysis, which utilized the Kyoto Encyclopedia of Genes and Genomes (KEGG) database.^3^

### 2.6 Statistical analysis

The data is presented as mean ± standard error of the mean (SEM). All data was verified for normality and variance homogeneity before statistical analysis. The Student’s *t*-test (IBM SPSS, v 21.0) was used to assess the differences in serum antioxidant, immune indicators levels, as well as fecal odorants and SIgA levels between the two groups on day 0 and 42. The non-parametric test was used to analyze the differences in fecal scores. When *p* < 0.05, the differences were regarded as significant.

## 3 Results

### 3.1 CPP did not affect physiological indicators

To determine that CPP was not detrimental to the dogs’ health, the blood biochemical analyses were performed on dogs fed for 42 days. The results showed that serum alanine aminotransferase (ALT), aspartate aminotransferase (AST), total bilirubin (TB), and glutamyl-transferase (GGT) remained within the normal physiologic range (Supplementary Table 1). In addition, the dogs’ body weight (Supplementary Figure 1, *p* > 0.05) and food intake (Supplementary Figure 2, *p* > 0.05) exhibited no significant changes throughout the study.

### 3.2 CPP enhanced serum antioxidant enzyme activity and lowered inflammatory factor levels

**FIGURE 1:**
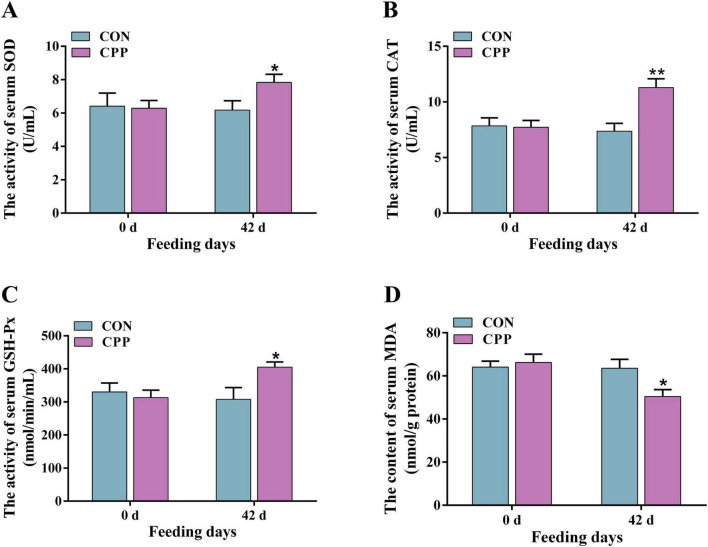
The activity changes of serum SOD **(A)**, CAT **(B)**, GSH-Px **(C)**, and MDA **(D)** level. *p < 0.05; **p < 0.01.

**FIGURE 2:**
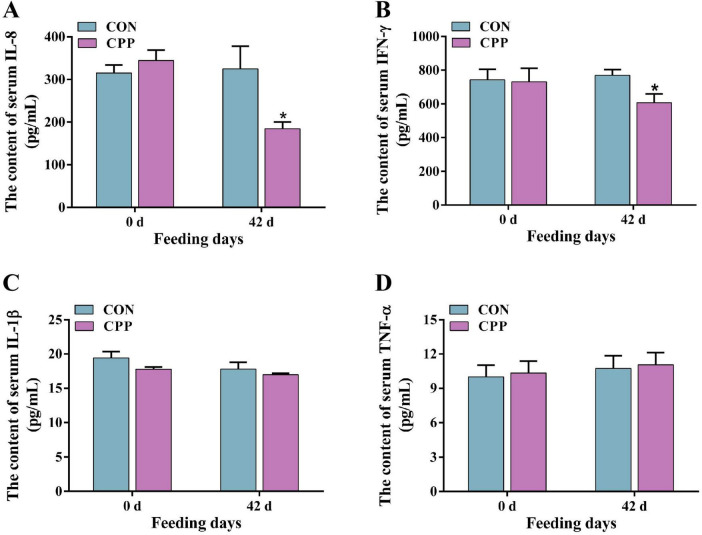
The changes in serum IL-8 **(A)**, IFN-γ **(B)**, IL-1β **(C)**, and TNF-α **(D)** level. *p < 0.05.

The results from biochemical and ELISA studies showed that feeding CPP for 42 days considerably enhanced the activity of canine serum SOD (*p* < 0.05) and GSH-Px (*p* < 0.05) (Figures 1A, C). Additionally, it significantly increased the CAT activity (Figure 1B, *p* < 0.01) compared to the CON group. Furthermore, there was a significant decrease in the serum MDA content in the CPP group (Figure 1D, *p* < 0.05). As shown in Figure 2, the content of IL-8 and IFN-γ decreased significantly after CPP feeding (Figures 2A, B, *p* < 0.05). However, no significant variation was observed in IL-1β and TNF-α between the two groups (Figures 2C, D, *p* > 0.05).

### 3.3 CPP reduced fecal H_2_S, 3-methylindole, NH_4_^+^-N content, and increased SIgA level

**FIGURE 3:**
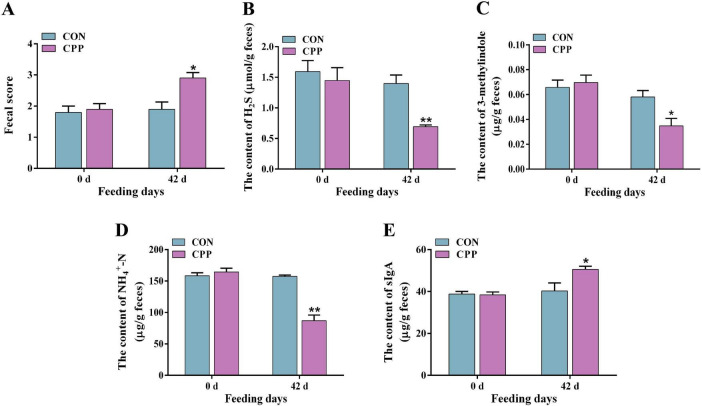
The fecal score **(A)** and content of fecal H_2_S **(B)**, 3-methylindole **(C)**, NH_4_^+^-N **(D)**, and SIgA **(E)**. *p < 0.05; **p < 0.01.

The fecal score, SIgA level, and the inhibitory effects of CPP on fecal odorants were estimated. The results demonstrated a significant increase in the fecal score of dogs following the administration of CPP for 42 days (Figure 3A, *p* < 0.05). Additionally, the levels of fecal odorants, including H_2_S (Figure 3B, *p* < 0.01), 3-methylindole (Figure 3C, *p* < 0.05), and NH_4_^+^-N (Figure 3D, *p* < 0.01), were significantly reduced. Moreover, a significant rise in the level of SIgA was observed (Figure 3E, *p* < 0.05).

### 3.4 CPP modulated the structural composition and abundance of intestinal microbiota

The impact of CPP on the intestinal microbiota of dogs was investigated using 16S rRNA sequencing. The sequencing results revealed that the Q30 value for all samples exceeded 93%. After filtering the raw tags, the proportion of effective tags that could be used for subsequent analysis was above 80% in all cases (Supplementary Table 2). This indicates that the sequencing data was reliable. As shown in Supplementary Figure 3, 165 OTUs were shared across two groups. Of these, 249 OTUs were unique to the CPP group and 230 OTUs to the CON group (Supplementary Figure 3A). All samples’ rarefaction curves eventually flatten out, suggesting that the number of species does not rise as the number of sequences does, and most of the microorganisms in each sample were detected sufficiently to characterize the sample’s flora (Supplementary Figures 3B, C). According to the rank abundance curve, CPP enhanced the species richness of the intestinal flora in dogs (Supplementary Figure 3D).

**FIGURE 4:**
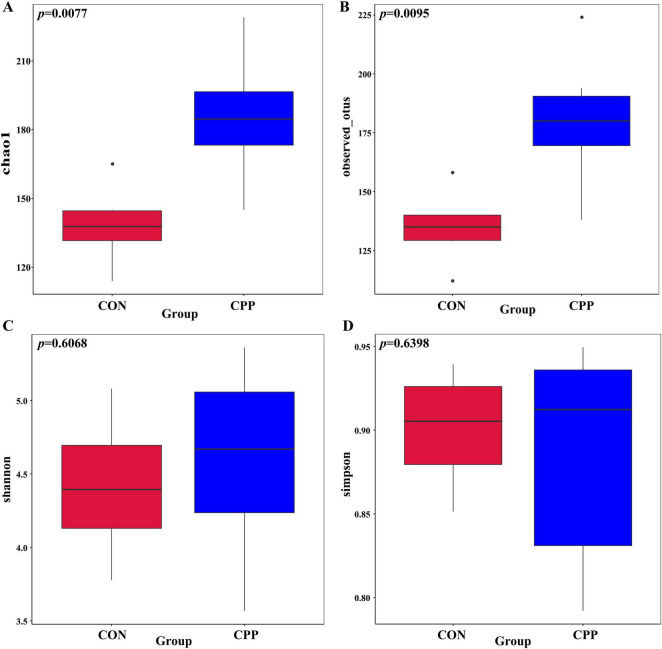
The Chao1 **(A)**, Observed_OTUs **(B)**, Shannon **(C)**, and Simpson **(D)** indexes in α-diversity analysis of intestinal flora.

**FIGURE 5:**
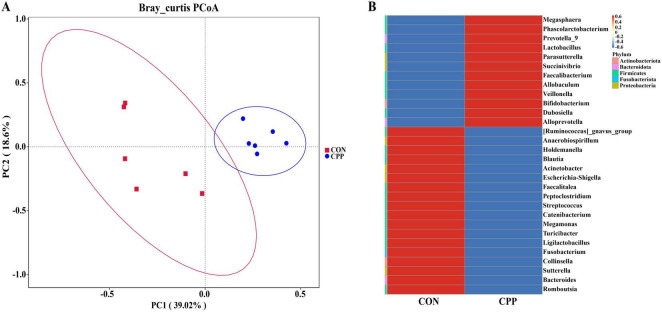
Principle coordinate analysis (PCoA) and relative abundance analysis of different groups. **(A)** PCoA score plot of dogs’ intestinal flora. **(B)** The genera with changed abundance between the two groups.

The microbiota richness of the CPP group was higher than that of the CON group, as shown by the significantly higher Chao1 and Observed_OTUs indexes (Figures 4A, B). In contrast, the Shannon and Simpson indexes did not show significant changes (Figures 4C, D). Additionally, principal coordinate analysis (PCoA) showed the sample distances between the CON and CPP groups and revealed that the CPP group was segregated from the CON group (Figure 5A). Figure 5B illustrates the main genera that changed in abundance between the two groups.

**FIGURE 6:**
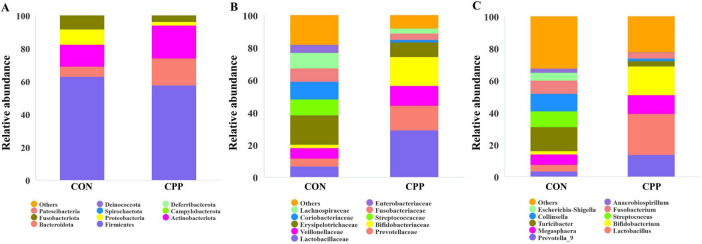
Composition of intestinal flora at the phylum **(A)**, family **(B)**, and genus **(C)** levels.

At the phylum level, Firmicutes, Bacteroidota, Fusobacteria, Actinobacteria, and Proteobacteria are the principal bacterial phylum in the dogs’ feces. Compared to the CON group, the CPP group had a decreased abundance of Firmicutes and Proteobacteria, with an increased abundance of Bacteroidota and Actinobacteriota (Figure 6A). This resulted in a 2.6-fold higher Bacteroidota/Firmicutes (B/F) ratio (Supplementary Figure 4). At the family level, the CPP group had a higher relative abundance of Bifidobacteriaceae, Veillonellaceae, Lactobacillaceae, and Prevotellaceae than the CON group. In contrast, the relative abundance of Coriobacteriaceae, Erysipelotrichaceae, and Streptococcaceae was lower (Figure 6B). At the genus level, CPP raised the relative abundance of Prevotella_9, Lactobacillus, Megasphaera, and Bifidobacterium and reduced the relative abundance of Turicibacter, Streptococcus, and Collinsella (Figure 6C).

**FIGURE 7:**
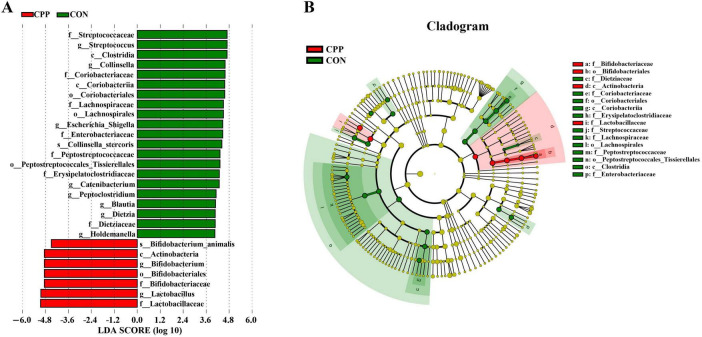
Linear discriminant analysis (LDA) effect size analysis of intestinal flora. **(A)** Biomarkers with LDA scores >4 are shown in the LDA value distribution histograms. **(B)** Cladogram showing a comparison of the microbial pro-file between the CON and CPP groups.

The microbial taxa that act as biomarkers for each group were found using the LEfSe analysis. As shown in Figure 7, Streptococcaceae, Streptococcus, Clostridia, Collinsella, Coriobacteriaceae, etc. were significantly enriched in the CON group, and Bifidobacterium*_*animalis, Actinobacteria, Bifidobacterium, Bifidobacteriales, Bifidobacteriaceae, Lactobacillus, and Lactobacillaceae were significantly enriched in the CPP group.

### 3.5 Predicted functional analysis by PICRUSt

**FIGURE 8:**
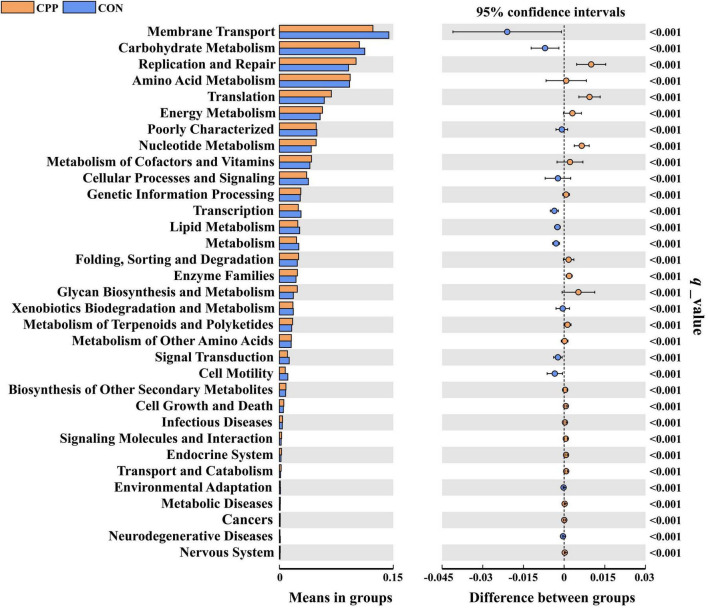
Predicted functions for the altered metagenome of gut microbiota between the CON and CPP groups (*q* < 0.01).

Analysis of significant differences in functions between the two groups revealed that 33 KEGG pathways were altered in the CPP group at level 2, among which 21 were increased and 12 decreased (Figure 8). The top 20 presumed microbial functions were compared using level 3 of the KEGG pathways (Table 3). Compared to the CON group, the CPP group had significantly higher abundance in the Genetic Information Processing-related pathways, such as “DNA repair and recombination proteins,” “Ribosome,” “Ribosome Biogenesis,” “Aminoacyl-tRNA biosynthesis,” “DNA replication proteins,” “Homologous recombination,” “Translation proteins,” and “Mismatch repair.” Moreover, Metabolism-related pathways were also found, including “Purine metabolism,” “Peptidases,” “Pyrimidine metabolism,” “Amino acid related enzymes,” “Cysteine and methionine metabolism,” and “Peptidoglycan biosynthesis.”

**TABLE 3:** The selected top 20 most abundant pathways at level 3.

KEGG pathways	Group		*q-*value
	**CON**	**CPP**	
Environmental information processing			
Transporters	0.0776 ± 0.004	0.0653 ± 0.011	0.042
Two-component system	0.0117 ± 0.001	0.0095 ± 0.001	0.019
Translation proteins	0.0094 ± 0.000	0.0099 ± 0.000	0.040
**Genetic information processing**			
DNA repair and recombination proteins	0.0294 ± 0.001	0.0334 ± 0.001	0.002
Ribosome	0.0242 ± 0.001	0.0295 ± 0.002	0.002
Transcription factors	0.0186 ± 0.001	0.0145 ± 0.001	0.002
Ribosome biogenesis	0.0148 ± 0.001	0.0157 ± 0.000	0.014
Aminoacyl-tRNA biosynthesis	0.0126 ± 0.001	0.0150 ± 0.001	0.005
DNA replication proteins	0.0123 ± 0.000	0.0135 ± 0.001	0.030
Homologous recombination	0.0097 ± 0.000	0.0106 ± 0.000	0.009
Mismatch repair	0.0083 ± 0.000	0.0092 ± 0.001	0.010
**Metabolism**			
Purine metabolism	0.0229 ± 0.001	0.0266 ± 0.001	0.002
Peptidases	0.0193 ± 0.001	0.0217 ± 0.001	0.008
Pyrimidine metabolism	0.0190 ± 0.000	0.0218 ± 0.002	0.009
Amino acid related enzymes	0.0149 ± 0.001	0.0170 ± 0.000	0.007
Methane metabolism	0.0119 ± 0.001	0.0110 ± 0.000	0.035
Fructose and mannose metabolism	0.0120 ± 0.001	0.0100 ± 0.001	0.009
Pyruvate metabolism	0.0108 ± 0.000	0.0089 ± 0.001	0.002
Cysteine and methionine metabolism	0.0096 ± 0.000	0.0105 ± 0.000	0.009
Peptidoglycan biosynthesis	0.0087 ± 0.000	0.0098 ± 0.001	0.009

### 3.6 Correlation analysis between differential genera and serum indicators

**FIGURE 9:**
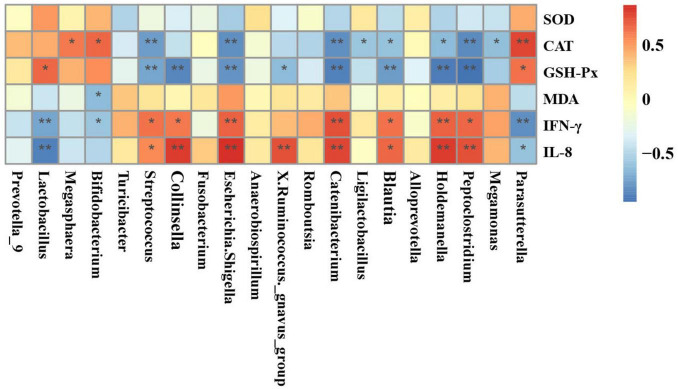
Heatmaps of Spearman’s correlation analysis between differential genera and serum indicators. Red and blue boxes represent positive and negative correlations, respectively. *p < 0.05; **p < 0.01.

Figure 9 demonstrates the results of Spearman’s correlation analysis, which examined the relationship between fecal differential microbial and serum indicators. The analysis revealed that the CAT showed a significant positive correlation with the Parasutterella, Bifidobacterium, and Megasphaera. On the other hand, it showed a negative correlation with the Megamonas, Peptoclostridium, Holdemanella, Blautia, Ligilactobacillus, Catenibacterium, Escherichia*-*Shigella, and Streptococcus. The GSH-Px was significantly positively correlated with the Parasutterella and Lactobacillus while negatively correlated with the Peptoclostridium, Holdemanella, Blautia, Catenibacterium, X. Ruminococcus_gnavus_group, Escherichia-Shigella, Collinsella, and Streptococcus. The MDA exhibited a significant negative correlation alone with the Bifidobacterium. The levels of IFN-γ and IL-8 were shown to have a strong positive correlation with the presence of Peptoclostridium, Holdemanella, Blautia, Catenibacterium, Escherichia-Shigella, Collinsella, and Streptococcus. Conversely, there was a negative correlation between the levels of IFN-γ and IL-8 and the presence of Parasutterella and Lactobacillus. Moreover, the IFN-γ was also significantly negatively correlated with the Bifidobacterium.

## 4 Discussion

This study investigates the effects of CPP on the health parameters and intestinal flora of dogs. The results revealed that CPP enhances dogs’ health by increasing serum antioxidant enzyme activity, reducing inflammatory factor levels, and regulating the composition of the intestinal flora. Oxidative stress poses a threat to both humans and animals and is linked to various chronic diseases, including cancer, neurological disorders, and cardiovascular disease (Pizzino et al., 2017). Therefore, the antioxidant properties protect the overall wellbeing of the organism and extend its lifespan. SOD, GSH-Px, and CAT are three primary antioxidant enzymes, while MDA serves as a biomarker of oxidative damage that can be detrimental to health (Tsikas, 2017). Prior research has shown that CPP can increase the levels and activity of serum GSH-Px and CAT while decreasing MDA levels in mice with liver damage (Chowdhury et al., 2015). Additionally, Qian et al. (2021) showed that feeding aqueous solution containing CPP causes a significant decrease in hepatic MDA content and a slight increase in SOD content in mice, although no significant changes were observed (Hu et al., 2021). Similarly, Chen et al. (2012) found that CPP effectively suppressed the rise in MDA levels in HepG2 cells caused by oxidative stress while simultaneously increasing the activity of CAT and GSH-Px enzymes, as well as the concentration of SOD. Similar to research conducted on other animal species, the current study demonstrated that supplementation of CPP resulted in significant rises in dogs’ blood SOD, GSH-Px, and CAT activities while reducing MDA levels. These findings indicate that CPP greatly enhances the antioxidant capacity in dogs.

Pro-inflammatory cytokines regulate intestinal mucosal barrier function and can increase mucosal epithelial cell permeability, consequently inducing inflammation (Katsanos and Papadakis, 2017). Studies conducted on mice with colitis have demonstrated that intact CPP is more efficacious in reducing the expression of pro-inflammatory factors IL-6, CXC motif chemokine ligand 2 (CXCL2), IL-1β, and TNF-α than a single CPP extract (Kawabata et al., 2018; Tinh et al., 2021). Han et al. observed a significant inhibition of serum IL-6 and TNF-α content in mice with high-fat-induced intestinal mucosal barrier damage following CPP supplementation (Han et al., 2023). It was found that CPP significantly reduced the serum content of IL-8 and IFN-γ, suggesting a potential contribution of CPP to the enhancement of intestinal mucosal defenses in dogs. However, no significant change in TNF-α and IL-1β levels was observed, which differed from other studies and may be attributed to species and diet differences. SIgA, found in high quantities in the mucous membranes of animals’ intestines, is crucial in protecting against the infiltration of harmful intestinal pathogens and toxic substances (Mantis et al., 2011). This study showed a significant increase in fecal SIgA content following CPP supplementation. This further underlines the beneficial effect of CPP on the intestinal health of dogs.

Chenpi powder has been proven to affect the composition of intestinal flora (Hu et al., 2021; Qian et al., 2021). However, there is a lack of research data pertaining to dogs. The 16S rRNA sequencing results revealed that the five predominant microbial phyla in dogs’ feces were Firmicutes, Bacteroidota, Fusobacteria, Actinobacteria, and Proteobacteria, consistent with findings from other studies (Honneffer et al., 2017; Pilla and Suchodolski, 2020). CPP supplementation increased the B/F ratio compared to the CON group. It has been shown that the B/F ratio significantly decreases under conditions of impaired intestinal health in obese mice (Evans et al., 2014; Long et al., 2019). Conversely, the addition of CPP inhibition to the diet has been discovered to prevent the reduction of fecal B/F ratio in obese mice (Li et al., 2021). Moreover, Qian et al. (2021) also found that CPP increased the B/F ratio in the feces of healthy mice. Hoffman et al. (2017) proposed that a decrease in the B/F ratio leads to the production of inflammatory factors by pathogenic bacteria. These results indicate that CPP supplementation could elevate the B/F ratio, thus improving the intestinal health status of dogs. The α-diversity and β-diversity analyses showed that CPP supplementation affected the abundance of intestinal flora in dogs. Bifidobacteriaceae, Lactobacillaceae, and Prevotellaceae are beneficial bacteria for animals that ferment carbohydrates to produce short-chain fatty acids, which boost the host immune response and provide energy for colonocytes, and is essential for intestinal health (Minamoto et al., 2015; Rivera-Chávez et al., 2016; Shen et al., 2018; Vazquez-Baeza et al., 2016; Zhang et al., 2021). Furthermore, research has shown that Lactobacillus and Bifidobacterium can safeguard the health of the intestines by counteracting the establishment of detrimental bacteria, therefore affecting inflammation (Guandalini, 2011; Kok and Hutkins, 2018; Konstantinov et al., 2008; van Baarlen et al., 2009). Li et al. (2019) found that Lactobacillus and Bifidobacterium also alleviated oxidative stress damage in mice. Ojo et al. (2019) showed that Lactobacillus was negatively correlated with serum MDA levels, reflecting the organism’s antioxidant capacity to some extent, and the current results are consistent with the existing studies. It was also found that several beneficial bacteria with increased abundance in the CPP group were positively correlated with antioxidant enzymes and negatively correlated with MDA and pro-inflammatory factors. In contrast, Escherichia-Shigella and Turicibacter were negatively correlated with antioxidant enzymes and positively correlated with MDA and pro-inflammatory factors. This indicates that these CPP-enriched beneficial bacteria may be crucial in enhancing antioxidant activity and boosting the immune system. Also, CPP elevated the prevalence of Veillonellaceae in the dogs’ intestinal tract. Research has demonstrated that the abundance of Veillonellaceae significantly decreases in the intestinal tract of dogs with enteritis (Suchodolski et al., 2012a), whereas Veillonellaceae abundance rises in healthy dogs following probiotic supplementation (Garcia-Mazcorro et al., 2017).

The results of KEGG pathway analysis showed that the enrichment of genetic information processing and metabolism-related pathways in the CPP group was significantly higher than that in the CON group, such as DNA repair and recombination proteins, aminoacyl-tRNA biosynthesis, amino acid metabolism, nucleotide metabolism, metabolism of cofactors and vitamins, energy metabolism, and peptidoglycan biosynthesis, suggesting that CPP has a higher level of nutrient metabolism, which is favorable for the reproduction of the intestinal flora.

The studies showed a strong link between the onset of colitis and specific pathogenic bacteria like Escherichia-Shigella and Turicibacter (Peng et al., 2020; Wan et al., 2021). Doulidis et al. (2023) indicated a significant increase in the abundance of intestinal Escherichia-Shigella in dogs with enteritis. Similarly, Díaz-Regañón et al. (2023) found a 16-fold increase in the abundance of Escherichia-Shigella in the intestinal tract of dogs with enteritis compared to healthy dogs. Additionally, an elevated presence of Enterobacteriaceae, which indicates an imbalance in the gut microbiota, was found to negatively correlate with beneficial metabolites in the intestine (Vazquez-Baeza et al., 2016). Several studies have shown a significant increase in intestinal Enterobacteriaceae abundance in dogs with inflammatory bowel disease (Suchodolski et al., 2010; Suchodolski et al., 2012a; Xenoulis et al., 2008). Furthermore, these bacteria could produce endotoxins, leading to systemic inflammation (Boopathi et al., 2023; Deng et al., 2023). Coriobacteriaceae and Fusobacteriaceae produce phenolic compounds posing a serious threat to host health (Saito et al., 2018). Coriobacteriaceae abundance was found to be approximately twice as high in dogs with enteritis as in healthy dogs (Díaz-Regañón et al., 2023). The obtained results of the present study suggested that CPP promoted intestinal and overall health in dogs.

The unpleasant odors from animal feces can be bothersome, impacting mood and sensory experiences. The primary odorous substances in dogs’ feces include sulfur compounds, indolic compounds, and ammonia, which result from the metabolic breakdown of food residues (Cho et al., 2015). Research has not yet investigated the decrease in the number of these malodorous compounds by CPP. This study uncovered that CPP significantly reduced the levels of H_2_S, NH_4_^+^-N, and 3-methylindole in dogs’ feces, indicating a favorable impact of CPP on odor reduction. This may be attributed to decreased odor-producing bacteria such as Escherichia-Shigella, Enterobacteriaceae, Streptococcus, and Fusobacteria (Carbonero et al., 2012; Richardson et al., 2013). However, further research is still needed to elucidate the specific regulatory mechanism.

## 5 Conclusion

In conclusion, this investigation found that CPP enhanced the antioxidant and immunological functions of dogs by augmenting the activity of antioxidant enzymes in the serum and reducing the levels of MDA and inflammatory indicators. Furthermore, CPP increased intestinal SIgA levels and successfully reduced the content of malodorous compounds in the feces, which contributed to improving intestinal immunity and alleviating fecal odor. CPP modulated the composition of the canine intestinal flora and stimulated beneficial bacterial growth, which had a considerable positive impact on the host’s intestinal and organismal health. These findings provide an innovative perspective and a scientific theoretical foundation for developing functional foods promoting pets’ health in the future.

## Data Availability

The datasets presented in this study can be found in online repositories. The names of the repository/repositories and accession number(s) can be found below: https://www.ncbi.nlm.nih.gov/, PRJNA1078076.

## Footnotes

1
http://ccb.jhu.edu/software/FLASH/

2
www.arb-silva.de/

3 https://www.genome.jp/kegg/pathway.html, accessed on 10 February 2023.
