# Supplementary material for: Effects of Chenpi (Citrus reticulata cv. Chachiensis) on serum antioxidant enzymes, inflammatory factors, and intestinal health in Beagle dogs
Source: Front Microbiol. 2025 Jan 7;15:1415860. doi: 10.3389/fmicb.2024.1415860 (PMC11747223; doi:10.3389/fmicb.2024.1415860)
Supplement: Supplementary file 1 [file Supplementary_file_1.docx]

Supplementary Material

**Supplementary Table S1**. The dogs’ serum biochemical parameters in CON and CPP group.

| **Feeding days** | **Group** | **Number** | **Items** | | | | | | |
| --- | --- | --- | --- | --- | --- | --- | --- | --- | --- |
|  |  |  | **GGT**  **(0-10 U/L)** | **ALT**  **(5-125 U/L)** | **AST**  **(0-50 U/L)** | **TB**  **(0-15 μmol/L)** | **SUN**  **(2.5-9.6 mmol/L)** | **SCR**  **(28-159 μmol/L)** | **PI**  **(0.81-2.19 mmol/L)** |
| 0 d | CON | 1 | < 2 | 81 | 37 | < 0.1 | 6.03 | 64.2 | 1.13 |
|  |  | 2 | < 2 | 114 | 58 | 19.2 | 4.21 | 42 | 0.94 |
|  |  | 3 | < 2 | 62 | 44 | < 0.1 | 4.65 | 54.4 | 0.99 |
|  |  | 4 | < 2 | 71 | 55 | < 0.1 | 6.33 | 66 | 1.17 |
|  |  | 5 | < 2 | 56 | 57 | < 0.1 | 6.22 | 47.8 | 1.10 |
|  |  | 6 | < 2 | 92 | 37 | < 0.1 | 6.04 | 64 | 1.16 |
|  |  | 7 | < 2 | 72 | 56 | < 0.1 | 4.23 | 44.9 | 2.38 |
|  |  | 8 | < 2 | 51 | 56 | 5 | 4.47 | 32.6 | 2.56 |
|  |  | 9 | < 2 | 70 | 46 | < 0.1 | 3.76 | 30.5 | 2.82 |
|  |  | 10 | < 2 | 45 | 38 | < 0.1 | 6.09 | 44.9 | 2.54 |
|  | CPP | 1 | 2 | 87 | 42 | 3.2 | 5.65 | 45.1 | 1.25 |
|  |  | 2 | < 2 | 69 | 43 | 3.9 | 4.53 | 44.3 | 1.12 |
|  |  | 3 | < 2 | 51 | 34 | < 0.1 | 5.15 | 40.7 | 2.37 |
|  |  | 4 | < 2 | 75 | 49 | < 0.1 | 4.01 | 38 | 2.54 |
|  |  | 5 | < 2 | 75 | 26 | < 0.1 | 4.31 | 43.3 | 1.07 |
|  |  | 6 | < 2 | 40 | 34 | < 0.1 | 5.15 | 40.8 | 2.52 |
|  |  | 7 | < 2 | 60 | 38 | < 0.1 | 4.66 | 33.5 | 3.09 |
|  |  | 8 | < 2 | 46 | 24 | < 0.1 | 4.03 | 29.2 | 2.54 |
|  |  | 9 | < 2 | 66 | 10 | 1.7 | 3.45 | 43.4 | 1.57 |
|  |  | 10 | < 2 | 50 | 42 | 8.3 | 4.6 | 44.7 | 1.23 |
| 42 d | CON | 1 | < 2 | 33 | 20 | 0.2 | 4.89 | 87.5 | 1.5 |
|  |  | 2 | < 2 | 53 | 24 | 2 | 4.69 | 74.8 | 1.51 |
|  |  | 3 | < 2 | 38 | 21 | 1.7 | 3.59 | 67.8 | 1.27 |
|  |  | 4 | < 2 | 33 | 24 | < 0.1 | 5.01 | 69.8 | 1.17 |
|  |  | 5 | < 2 | 48 | 32 | < 0.1 | 4.53 | 67.5 | 1.31 |
|  |  | 6 | < 2 | 43 | 42 | 2.4 | 4.34 | 89.4 | 1.12 |
|  |  | 7 | < 2 | 39 | 16 | < 0.1 | 4.36 | 79.1 | 1.35 |
|  |  | 8 | < 2 | 35 | 24 | 3.2 | 4.32 | 84.5 | 2.08 |
|  |  | 9 | < 2 | 33 | 20 | 0.7 | 4.3 | 60.9 | 2.14 |
|  |  | 10 | < 2 | 60 | 24 | 5.3 | 3.93 | 47.9 | 1.25 |
|  | CPP | 1 | 3 | 41 | 16 | 0.5 | 3.17 | 41.5 | 1.71 |
|  |  | 2 | < 2 | 43 | 29 | 1.2 | 3.23 | 41.2 | 2.57 |
|  |  | 3 | < 2 | 62 | 16 | 1.1 | 4.08 | 44.3 | 1.74 |
|  |  | 4 | < 2 | 39 | 18 | 1.2 | 3.4 | 49.6 | 1.22 |
|  |  | 5 | < 2 | 42 | 11 | 0.7 | 3.52 | 49.4 | 2.05 |
|  |  | 6 | < 2 | 34 | 16 | 0.4 | 2.85 | 36 | 2.44 |
|  |  | 7 | < 2 | 40 | 33 | 0.9 | 5.38 | 52.7 | 2.46 |
|  |  | 8 | < 2 | 35 | 23 | 0.9 | 3.73 | 72.5 | 1.85 |
|  |  | 9 | < 2 | 82 | 13 | 0.5 | 3.25 | 57.3 | 1.77 |
|  |  | 10 | < 2 | 63 | 8 | 0.5 | 3.86 | 41.3 | 1.73 |

Note: GGT, ALT, AST, TB, SUN, SCR and PI represent glutamyltransferase, alanine aminotransferase, aspartate aminotransferase, total bilirubin, serum urea nitrogen, serum creatinine and inorganic phosphorus, respectively.

**Supplementary Table S2.** The statistical results of 16S rRNA sequencing data.

| **Group** | **Sample** | **Raw reads** | **Effective tags** | **GC (%)** | **Q30 (%)** | **Effective (%)** |
| --- | --- | --- | --- | --- | --- | --- |
| CON | CON1 | 101611 | 96412 | 55.91 | 94.75 | 95 |
|  | CON2 | 111463 | 92035 | 53.41 | 93.41 | 83 |
|  | CON3 | 115675 | 103856 | 52.81 | 94.16 | 90 |
|  | CON4 | 99554 | 92769 | 52.08 | 94.09 | 93 |
|  | CON5 | 112464 | 105483 | 53.67 | 94.42 | 94 |
|  | CON6 | 102488 | 93881 | 55.79 | 94.08 | 92 |
| CPP | CPP1 | 105835 | 91357 | 55.17 | 93.98 | 86 |
|  | CPP2 | 110400 | 92960 | 55.13 | 94.51 | 84 |
|  | CPP3 | 117389 | 108325 | 55.13 | 94.70 | 92 |
|  | CPP4 | 105165 | 97097 | 53.69 | 94.96 | 92 |
|  | CPP5 | 103822 | 97413 | 54.03 | 94.70 | 94 |
|  | CPP6 | 107554 | 101656 | 52.94 | 94.26 | 95 |

**
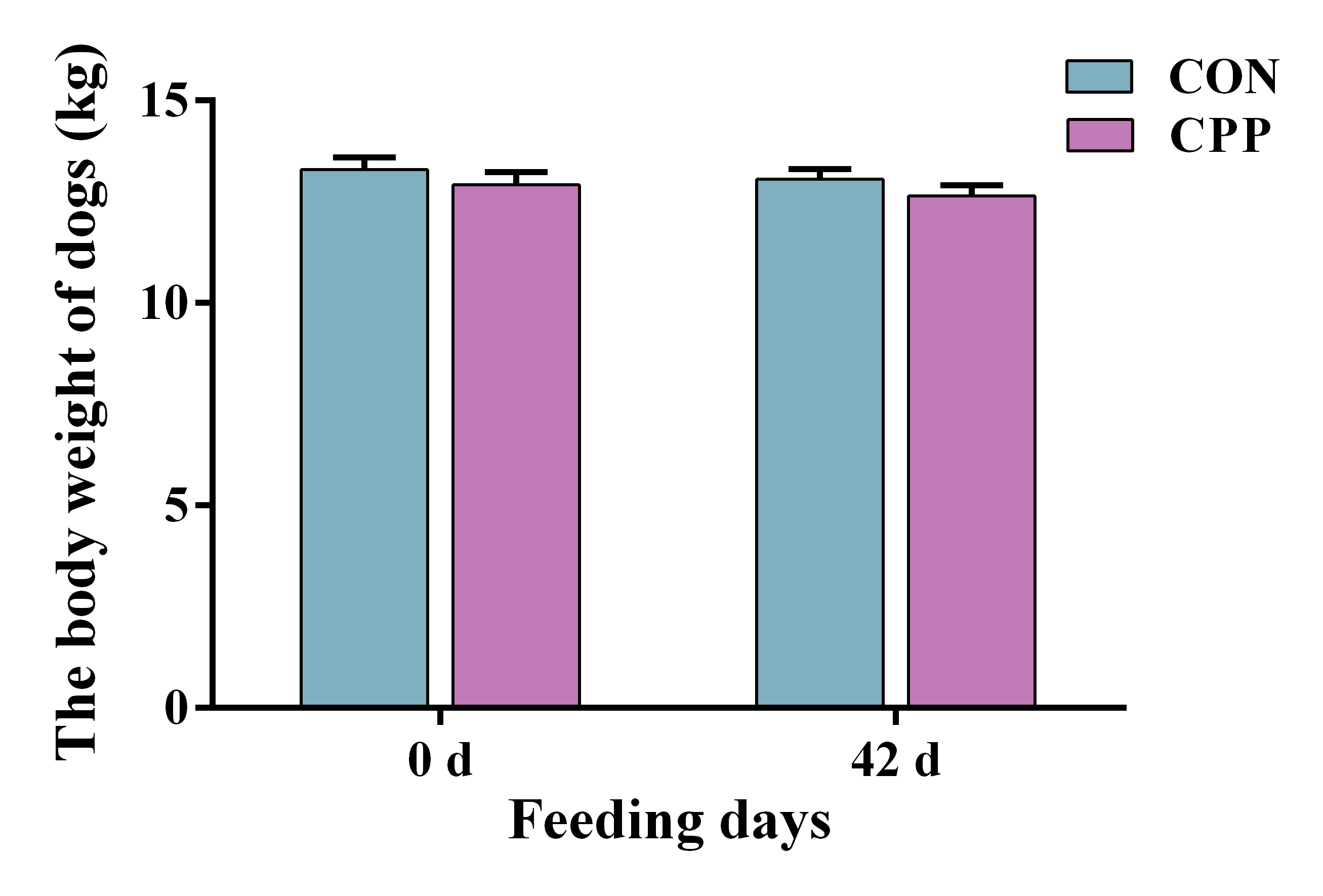
**

**Supplementary Figure S1**. The body weight of dogs during the experiment.


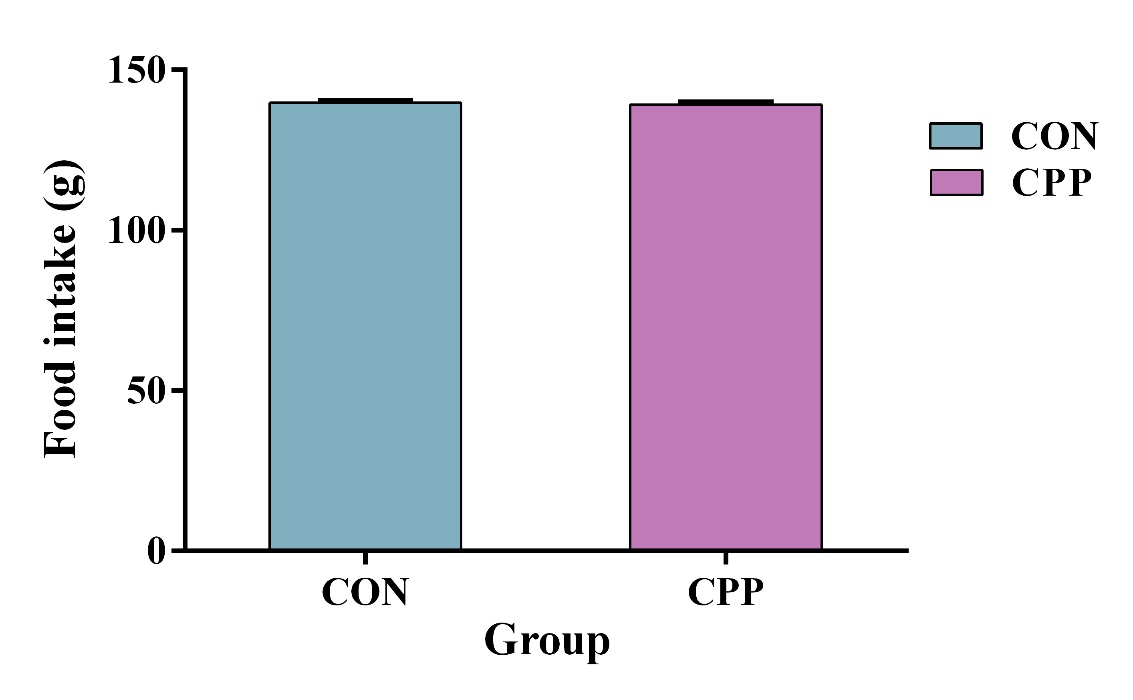


**Supplementary Figure S2**. The food intake of dogs during the experiment

**
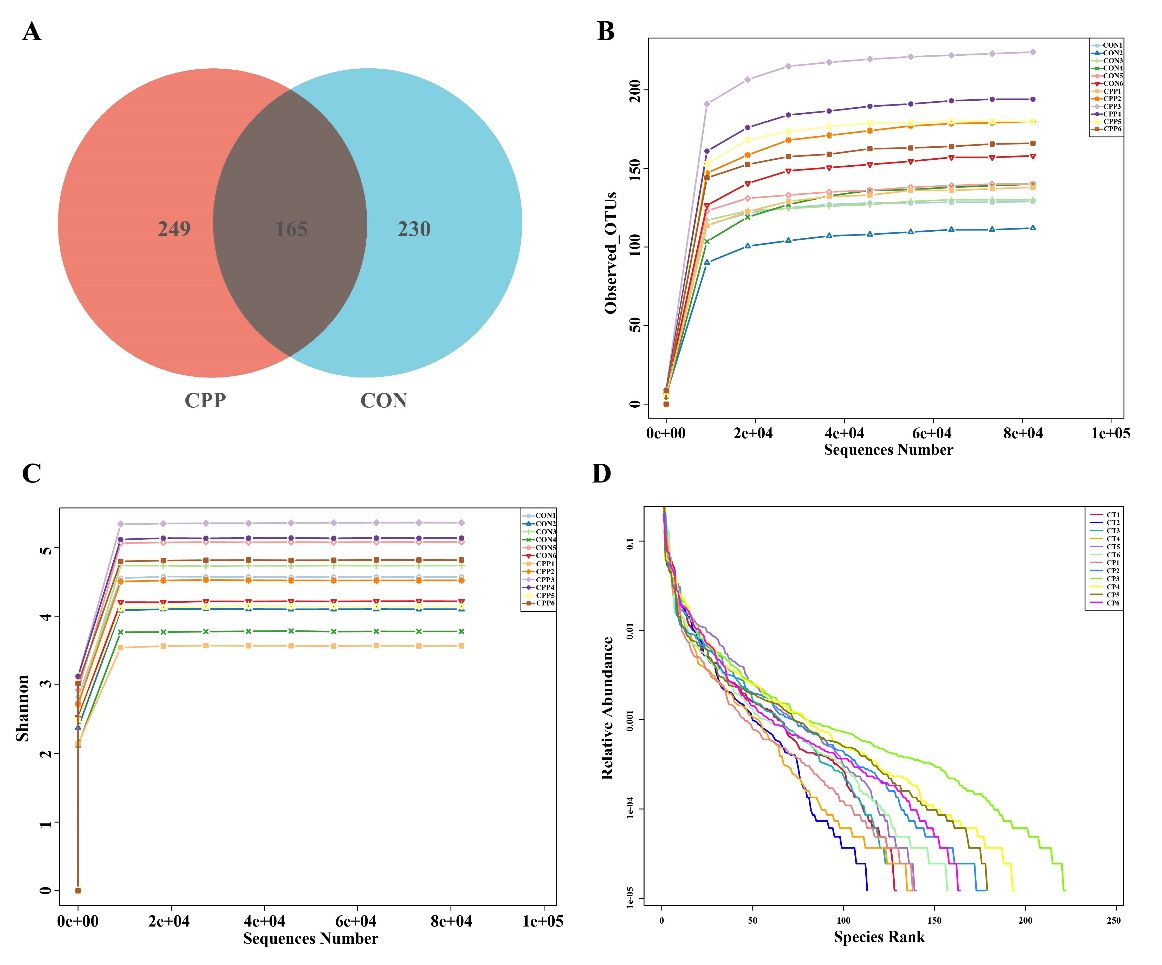
**

**Supplementary Figure S3**. Summary of 16S rRNA sequencing data. (A) Venn diagram of shared or unique OTUs in CON and CPP group. (B, C) The rarefaction curves of samples. (D) The rank abundance curve of samples.


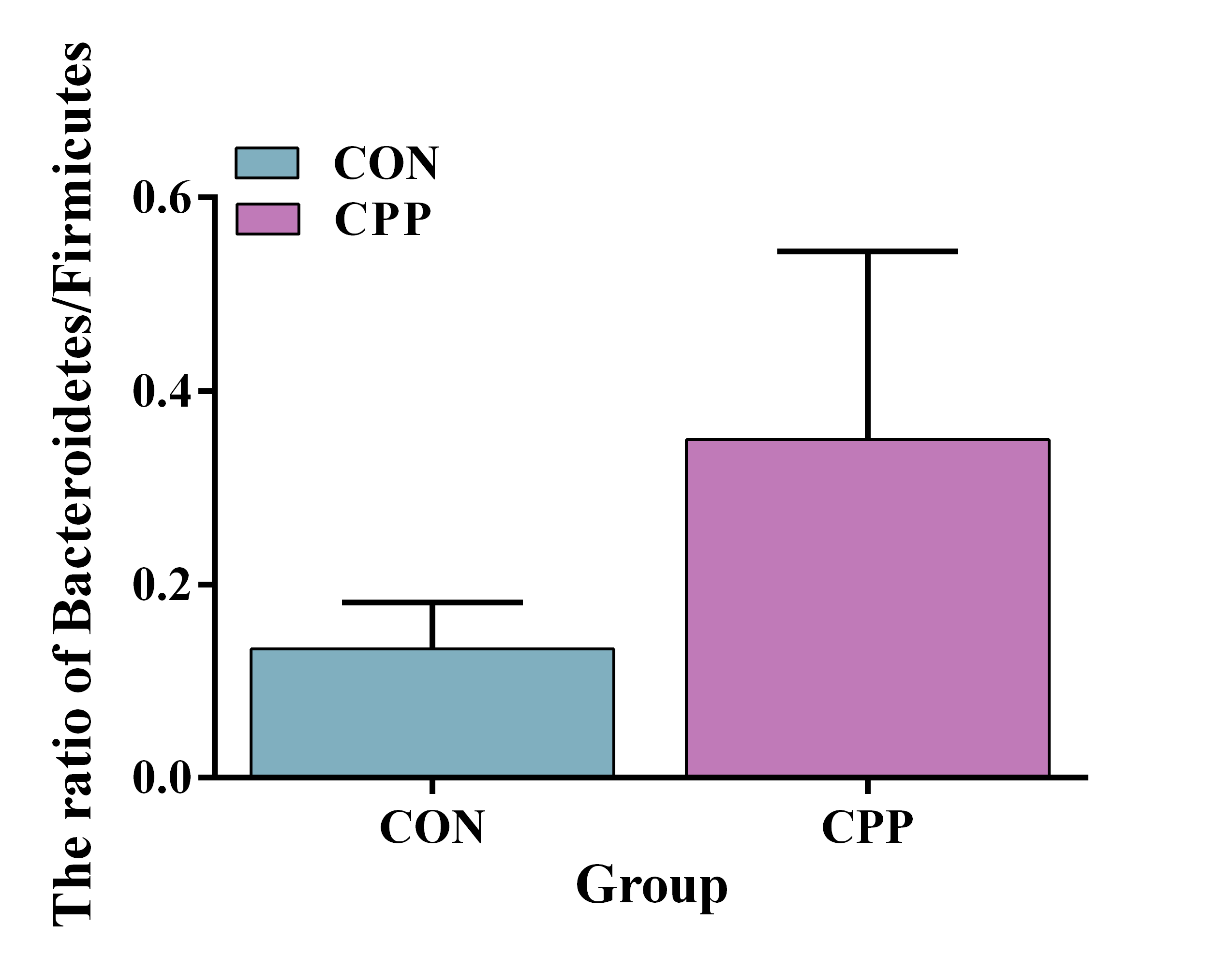


**Supplementary Figure S4**. The ratio of *Bacteroidota*/*Firmicutes* in CON and CPP group.
